# Heat of Oxidation of Aqueous Sulfur Dioxide With Gaseous Chlorine

**DOI:** 10.6028/jres.067A.044

**Published:** 1963-10-01

**Authors:** Walter H. Johnson, John R. Ambrose

## Abstract

The heat of oxidation of aqueous sulfur dioxide with gaseous chlorine has been determined by calorimetric methods. The results correspond to the reaction:

$$\begin{array}{c} {\text{Cl}}_{2}\left(g\right)+{\text{SO}}_{2}\cdot 2500\;H_{2}O+2502\;H_{2}O=H_{2}{\text{SO}}_{4}\cdot 2500\;H_{2}O+2\left(\text{HCl}\cdot 1250\;H_{2}O\right),\\ \Delta H\left(25{}^{\circ}C\right)=-77.28\pm 0.14\;\text{kcal}/\text{mole}. \end{array}$$

The heat of formation of aqueous sulfuric acid (in 2500 moles of water) has been calculated to be −213.92 kcal/mole.

## 1. Introduction

An investigation of the heats of solution and oxidation of sulfur dioxide to sulfuric acid using bromine as the oxidizing substance was recently made at the University of Lund, Sweden [l].^2^ Although the data on the heats of solution of sulfur dioxide were in substantial agreement with the previous data [2] the calculated heat of formation of sulfuric acid differed by approximately 0.3 kcal/mole from the results obtained in several recent determinations [3, 4, 5, 6]. Because the heat of formation of sulfuric acid seems to be well substantiated it seemed probable that the discrepancy must be attributed to the data on the heat of formation of hydrobromic acid. It seemed desirable, therefore, to make a new determination of the heat of oxidation of sulfur dioxide using chlorine as the oxidizing agent. The combined data would be indicative of the consistency between the data on hydrochloric and hydrobromic acid as well as that between the data on sulfur dioxide and sulfuric acid. An error of 0.15 kcal/mole in the heat of formation of hydrobromic acid could account for the apparently high value obtained for the heat of formation of sulfuric acid obtained from the heat of oxidation of sulfur dioxide with bromine.

## 2. Materials

### 2.1. Iodine Solution

The 0.1 *N* iodine solution was prepared by dissolving 12.7 g of resublimed iodine in a concentrated solution containing 80 g of iodate-free potassium iodide and diluting to 2 liters. The solution was protected from light by covering the bottle with aluminum foil. Removal of the solution from the bottle was by means of a siphon. The solution was standardized against arsenious oxide, NBS standard sample 83b. Weight burets were used for all titrations.

### 2.2. Thiosulfate Solution

The 0.05 *N* sodium thiosulfate solution was prepared by adding 25 g of reagent-grade sodium thiosulfate pentahydrate and 0.2 g of sodium carbonate to 2 liters of water. The concentration of the solution was determined by comparison with the 0.1 *N* iodine solution.

### 2.3. Sulfur Dioxide Solution

A mass-spectrometric analysis of the gaseous sulfur dioxide was performed by E. E. Hughes of the Applied Analytical Research Section of the Analytical and Inorganic Chemistry Division. The analysis gave the following results in mole percent: 98 SO_2_, 1.3 CO, 0.4 N_2_, and 0.2 CS_2_. The 0.15 *M* sulfur dioxide solution was prepared by passing the gas through 4 liters of distilled water from which air and CO_2_ had been removed by boiling. A slight excess of SO_2_ was added. This excess was later removed by passing a stream of nitrogen through the solution while stirring which also removed most of the CO and CS_2_ impurities. The solution was protected from light by covering the bottle with aluminum foil. A slight pressure of nitrogen was maintained over the solution; removal from the bottle was by means of a siphon. The concentration of the solution was determined by adding a 50 ml portion to an excess of standard iodine solution and titrating the excess iodine with sodium thiosulfate solution.

### 2.4. Chlorine

The chlorine was taken from a cylinder of gas certified by the manufacturer to have a purity of not less than 98 percent. Noncondensible gases were removed by pumping with the cylinder immersed in liquid nitrogen. Before each calorimetric experiment about a liter of gas was removed which tended to reduce the amount of any volatile impurities.

### 2.5. Sulfuric and Hydrochloric Acid Solutions

The solutions of sulfuric and hydrochloric acid were prepared from reagent-grade materials and sealed into weighed sample bulbs. The concentrations were determined by breaking some of the bulbs into water and titrating with standard alkali solution.

## 3. Apparatus

The glass calorimeter was, except for minor changes, the same as that previously described [7]. The Teflon bearings were removed and the stirrer shaft was supported by ball bearings placed above the calorimeter head. An O-ring seal was fitted on the head to prevent leakage of gas around the stirrer shaft. A capillary inlet tube replaced the usual bulb-crushing apparatus. This tube passed through the calorimeter head and terminated at a point about 1 cm above the screw-type propeller. A capillary exit tube projected through the calorimeter head into the vapor space above the solution. Although the metal parts were not in contact with the solution, they were coated with paraffin to prevent corrosion by the moist vapors.

Calorimeter temperatures were measured to ±0.00005 °C by means of a glass-sheathed platinum resistance thermometer, a G-2 Mueller bridge and a high-sensitivity galvanometer. Timing of the experiments was by reference to the NBS standard second signals.

The calorimeter system was calibrated by means of electrical energy using a method which has been described [8] except that a more precise method was used to determine the time of the heating interval, and the source of the electrical energy was a constant voltage electronic power supply which was fed by a saturation transformer. During the heating period a small current passed through a 60,000-ohm resistance to ground. Upon closing the heater circuit, the positive pulse, resulting from the increase in potential across a 10,000-ohm portion of the 60,000-ohm resistor, served to trigger the start-channel of an electronic gating counter which was connected to a 10 kc standard frequency. When the heater circuit was opened the resulting negative pulse served to trigger the stop-channel of the counter. It was possible with this apparatus to determine the duration of the heating period to ±0.0001 sec, although a precision of ±0.01 sec would have been adequate.

The calorimeter was modified slightly for determination of the heats of mixing of the hydrochloric and sulfuric acids in the final sulfur dioxide solution. This involved the substitution of a bulb-crushing apparatus for the gas-inlet tube and the closing of the exit tube.

## 4. Unit of Energy and Atomic Weights

The unit of energy used in these calculations is the absolute joule obtained as the product of absolute volts, absolute amperes and mean solar seconds. The conventional thermochemical calorie is taken as equal to 4.1840 absolute joules. All atomic weights have been taken from the 1961 table of international atomic weights based on carbon-12 [9].

## 5. Procedure

One hundred cubic centimeters of 0.15 *M* sulfur dioxide were added to 400 cm^3^ of air-free water (at 22 °C) in the calorimeter vessel which was then covered and weighed. The calorimeter was then assembled, placed in a thermostat, the glass thermometer was inserted and the stirrer attached. The thermostat was adjusted to 25.0 °C and regulated within ±0.001 °C; the stirring rate was approximately 500 rpm by a reducing pulley from a synchronous motor. Nitrogen was passed through the inlet tube at about 100 cm^3^/min for about 5 min to remove the bulk of the oxygen in the air space in the calorimeter. The calorimeter was then preheated to about 23.5 °C and approached a steady thermal state after a drift-period of about 15 min. Calorimeter temperatures were then determined at 2-min intervals during the initial rating period. Chlorine was then introduced through the inlet tube at about 30 cm^3^/min until the desired calorimeter temperature (25 °C) was reached; the flow of chlorine was then interrupted and the connecting tube was flushed with nitrogen. During the experiment the exit tube was connected to a bubbling vessel which contained a measured quantity of standard iodine solution; a slight positive pressure (1 mm) was thus maintained in the calorimeter vessel. Calorimeter temperatures were observed at 1-min intervals during the reaction period and were continued at 2-min intervals during a final 20-min rating period with the calorimeter in a steady thermal state.

Aliquot portions of the resulting calorimetric solution were taken to determine the final concentration of sulfur dioxide by titration with iodine and the quantity of chloride by precipitation as silver chloride. The quantity of sulfur dioxide in the bubbling vessel was determined by titration of the excess iodine with standard thiosulfate solution. The initial concentration of sulfur dioxide was calculated from these data.

Two series of experiments were performed to determine the heats of mixing of the components in the final solution resulting from the reaction of chlorine with aqueous sulfur dioxide. The first series was to determine the heat of mixing of aqueous hydrochloric acid with the aqueous sulfur dioxide. The second series involved the heat of mixing of aqueous sulfuric acid with the sulfur dioxide-hydrochloric acid solution. The procedure differed somewhat in the heat-of-mixing experiments from that used in the sulfur dioxide-chlorine experiments in that each experiment was preceded by a calibration. This was possible since the temperature rise in each case was only about 0.05 °C. The calibration consisted of an initial rating period, a heating period, and a final rating period. The final rating period of the calibration then became the initial rating period of the heat-of-mixing experiment. At the end of this period the sample bulb was broken; since the temperature effect was complete within 30 sec, no separate reaction period was required and the subsequent temperature observations comprised the final rating period. The corrected temperature rise was determined by extrapolation of the initial and final time-temperature curves according to the method of Dickinson [10]. The concentration of sulfur dioxide in the final solution was determined in each case by titration with iodine solution.

## 6. Results and Discussion

The results of the electrical calibration experiments on the calorimetric system used for the heat of reaction of chlorine with aqueous sulfur dioxide are given in table 1. Δ*Rc* is the corrected temperature rise of the calorimetric system [11] as measured on the platinum thermometer; *E_e_* is the quantity of electrical energy introduced into the system; Δ*E_s_* is the deviation in the heat capacity of the actual system from that of the “standard” system as calculated from the mass of solution; and *E_s_* is the energy equivalent of the “standard” calorimeter system. The uncertainty interval has been taken as twice the standard deviation of the mean.

The results of the experiments on the reaction of gaseous chlorine with the aqueous sulfur dioxide solution are given in table 2. The heat evolved in the calorimetric process, *q*, is obtained as the product of Δ*Rc* and (*E_s_+*Δ*E_s_*). The column designated SO_2_(f) gives the analytically determined quantity of SO_2_ remaining in the final calorimetric solution. The column designated SO_2_(g) gives the quantity of SO_2_ carried from the calorimeter by flushing with nitrogen during the reaction period. The quantity *q*(dil) is a correction applied to make the concentrations of the individual components in the actual final solution correspond to the concentrations given in reaction (1). It includes corrections for the heats of vaporization of the sulfur dioxide and water carried out of the solution by flushing with nitrogen. The quantity of reaction was determined by the amount of chlorine, determined analytically as chloride, in the final calorimetric solution. The heat of reaction given in table 2 corresponds to the process:

$$\begin{array}{l} {\text{Cl}}_{2}\left(g\right)+\left(1.560\;{\text{SO}}_{2}+2964\;H_{2}O\right)\\ \;=\left[2\;\text{HCl}+H_{2}{\text{SO}}_{4}+0.560\;{\text{SO}}_{2}+2962\;H_{2}O\right]\\ \;\Delta H\left(25{}^{\circ}C\right)=-76.81\pm 0.13\;\text{kcal}/\text{mole}. \end{array}$$ (1)

The assigned uncertainty has been taken as the square root of the sum of the squares of the uncertainty intervals for the calibration experiments, the reaction experiments, and estimated values for all other known sources of error.

The results of the experiments on the heat of mixing of aqueous hydrochloric acid with aqueous sulfur dioxide are given in table 3. The energy due to the crushing of the sample bulb was shown through separate experiments to be completely negligible. The heat of solution given in table 3 corresponds to the process:

$$\begin{array}{l} \text{HCl}\cdot 6.708\;H_{2}O+\left(0.201\;{\text{SO}}_{2}+1577\;H_{2}O\right)\\ \;=\left[\text{HCl}+0.201\;{\text{SO}}_{2}+1583.7\;H_{2}O\right]\\ \;\Delta H\left(25{}^{\circ}C\right)=-1.625\pm 0.02\;\text{kcal}/\text{mole}. \end{array}$$ (2)

The results of the experiments on the heat of mixing of aqueous sulfuric acid with the hydrochloric acid-sulfur dioxide solution are given in table 4. The resulting heat of solution corresponds to the process:

$$\begin{array}{l} H_{2}{\text{SO}}_{4}\cdot 11.37\;H_{2}O+\left[0.386{\text{SO}}_{2}+1.423\;\text{HCl}+3033.2\;H_{2}O\right]\\ \;=\left[H_{2}{\text{SO}}_{4}+1.423\;\text{HCl}+0.386\;{\text{SO}}_{2}+3044.6\;H_{2}O\right]\\ \;\Delta H\left(25{}^{\circ}C\right)=-2.57\pm 0.04\;\text{kcal}/\text{mole}. \end{array}$$ (3)

The consistency of the data in tables 3 and 4 indicate that small differences in concentration do not affect the results beyond the limits of experimental error. The results given for eqs (1), (2), and (3) were therefore corrected to the stoichiometry specified in eqs (4), (5), and (6) by assuming that in each case only the reference component was present in water. Equations (1), (2), and (3) were combined with the heats of dilution of sulfur dioxide [1], sulfuric acid [2, 12], and hydrochloric acid [2], to obtain the processes:

$$\begin{array}{l} {\text{Cl}}_{2}\left(g\right)+2\left({\text{SO}}_{2}\cdot 2500\;H_{2}O\right)+2502\;H_{2}O\\ =\left[H_{2}{\text{SO}}_{4}\cdot 2500\;H_{2}O+2\left(\text{HCl}\cdot 1250\;H_{2}O\right)+{\text{SO}}_{2}\cdot 2500\;H_{2}O\right]\\ \;\Delta H\left(25{}^{\circ}C\right)=-75.92\pm 0.13\;\text{kcal}/\text{mole}\;{\text{Cl}}_{2}. \end{array}$$ (4)

$$\begin{array}{l} 2\left(\text{HCl}\cdot 1250\;H_{2}O\right)+\left({\text{SO}}_{2}\cdot 2500\;H_{2}O\right)\\ \;=\left[2\left(\text{HCl}\cdot 1250\;H_{2}O\right)+{\text{SO}}_{2}\cdot 2500\;H_{2}O\right]\\ \;\Delta H\left(25{}^{\circ}C\right)=0.25\pm 0.02\;\text{kcal}/\text{mole}\;\text{HCl}. \end{array}$$ (5)

$$\begin{array}{c} H_{2}{\text{SO}}_{4}\cdot 2500\;H_{2}O+\left[{\text{SO}}_{2}\cdot 2500\;H_{2}O+2\left(\text{HCl}\cdot 1250\;H_{2}O\right)\right]\\ =[H_{2}{\text{SO}}_{4}\cdot 2500\;H_{2}O+{\text{SO}}_{2}\cdot 2500\;H_{2}O\\ +2\left(\text{HCl}\cdot 1250\;H_{2}O\right)]\\ \Delta H\left(25{}^{\circ}C\right)=0.86\pm 0.04\;\text{kcal}/\text{mole}\;H_{2}{\text{SO}}_{4}. \end{array}$$ (6)

The appropriate combination of eqs (4), (5), and (6) yields the process:

$$\begin{array}{l} {\text{Cl}}_{2}\left(g\right)+{\text{SO}}_{2}\cdot 2500\;H_{2}O+2502\;H_{2}O\left(\text{liq}\right)\\ \;=H_{2}{\text{SO}}_{4}\cdot 2500\;H_{2}O+2\left(\text{HCl}\cdot 1250\;H_{2}O\right)\\ \;\Delta H\left(25{}^{\circ}C\right)=-77.28\pm 0.14\;\text{kcal}/\text{mole}. \end{array}$$ (7)

The heat of formation of aqueous sulfuric acid may be obtained from eq (7) together with data on the heats of formation of water and of aqueous hydrochloric acid [2], and the heat of formation of aqueous sulfur dioxide [1]:

$$\begin{array}{l} S_{\text{rhombic}}+2O_{2}\left(g\right)+H_{2}\left(g\right)+2500\;H_{2}O\\ \;=H_{2}{\text{SO}}_{4}\cdot 2500\;H_{2}O\\ \;\Delta H\left(25{}^{\circ}C\right)=-213.92\pm 0.17\;\text{kcal}/\text{mole}. \end{array}$$ (8)

The heat of formation of aqueous sulfuric acid was determined in a similar manner by the reaction of liquid bromine with aqueous sulfur dioxide [1] and −214.20 ±0.11 kcal/mole was obtained for the process corresponding to eq (8). The heat of formation of H_2_SO_4_·115 H_2_O has been determined by several independent calorimetric determinations using rotating-bomb techniques [3, 4, 5, 6]; the mean of these determinations gives the heat of formation as −212.20 ±0.10 kcal/mole and it appears that this value is well established. By taking 1.81 kcal/mole for the heat of dilution [2] the value obtained for the heat of formation of H_2_SO_4_·2500 H_2_O is −214.01 kcal/mole. The excellent agreement of the present investigation with this value would seem to indicate a high degree of consistency in the data on HCl, SO_2_, and H_2_SO_4_. The discrepancy of 0.33 kcal obtained in the results with bromine [1] may possibly be due to an error in the heat of formation of aqueous hydrobromic acid.

The combination of eq (7) with the data of Johnson and Sunner [1] yields:

12Cl2(g)+HBr⋅1250H2O=12Br2(liq)+HCl⋅1250H2OΔH(25°C)=−10.90kcal/mole. (9)

By taking −68.3149 kcal/mole for the heat of formation of water [2], −79.90 for the heat of formation of SO_2_·2500 H_2_O [1], and −214.01 for the heat of formation of H_2_SO_4_·2500 H_2_O we calculate −39.90 kcal/mole for the heat of formation of HCl·1250 H_2_O according to eq (7). This is in excellent agreement with the selected “best” value of −39.92 kcal/mole [2]. The substitution of this value in eq (9) gives −29.00 kcal/mole for the heat of formation of HBr·1250 H_2_O whereas the selected “best” value is given as −28.81 kcal/mole [2] and indicates a discrepancy of 0.19 kcal.

Sunner [13] is conducting an investigation of the heat of reaction of chlorine with aqueous arsenious oxide. The results of that investigation together with a similar determination using bromine [14] should give additional evidence regarding the consistency between the data on hydrogen bromide and hydrogen chloride.

## Figures and Tables

**Table 1:** Results of the electrical calibration experiments

Experiment	Δ*Rc*	*E_e_*	Δ*E_s_*	*E_s_*
				
	*Ohm*	*j*	*j/ohm*	*j/ohm*
1	0.160953	3713.03	+2.1	23071.1
2	.133728	3085.40	+3.7	23075.9
3	.099719	2301.69	+0.4	23082.2
4	.125095	2886.83	0.0	23077.1
5	.124301	2868.88	−0.4	23079.7
6	.123887	2858.87	+1.2	23077.6
	
Mean				23077.3
Standard deviation of the mean				±1.53

**Table 2:** Results of the experiments on the reaction of chlorine with aqueous sulfur dioxide

Experiment	Δ*Rc*	Δ*E_s_*	SO_2_(f)	SO_2_(g)	*q*(dil)	Cl_2_	−ΔH(25 °C)
							
	*Ohm*	*j/ohm*	*m moles*	*m mole*	*j*	*m moles*	*kj/mole*
1	0.153058	−1.1	3.156	0.0030	+8.03	11.006	321.64
2	.136404	0.0	4.107	.0058	−0.17	9.796	321.32
3	.126626	−0.2	5.845	.0025	+2.18	9.074	322.28
4	.107620	+0.5	7.210	.0028	+9.12	7.761	321.18
5	.121110	−0.1	5.891	.0034	+2.51	8.733	320.32
6	.132806	−0.9	5.094	.0032	+2.18	9.536	321.61
	
Mean							321.39
Standard deviation of the mean							±0.26

**Table 3:** Results of the experiments on the heat of mixing of aqueous hydrochloric acid with aqueous sulfur dioxide

Experiment	Δ*Rc*	*E*	*q*	*q*(dil)	HCl(aq)	−ΔH(25 °C)
						
	*Ohm*	*j/ohm*	*j*	*j*	*m moles*	*kj/mole*
1	0.005661	23190	131.28	+0.32	19.216	6.85
2	.005538	23155	128.23	+0.16	18.508	6.94
3	.004798	23210	111.36	−0.21	16.485	6.74
4	.005132	23201	119.07	0.00	17.610	6.76
5	.004987	23208	115.74	−0.14	17.072	6.77
6	.004720	23176	109.39	−0.27	16.204	6.74
	
Mean						6.80
Standard deviation of the mean						±0.03

**Table 4:** Results of the experiments on the heat of mixing of aqueous sulfuric acid with the aqueous sulfur dioxide-hydrochloric acid solution

Experiment	Δ*Rc*	*E_a_*	*q*	*q*(dil)	H_2_SO_4_(aq)	−ΔH(25 °C)
						
	*Ohm*	*j/ohm*	*j*	*j*	*m moles*	*kj/mole*
1	0.004509	23169	104.47	+2.85	9.771	10.98
2	.004044	23171	93.70	−2.18	8.647	10.58
3	.004286	23161	99.27	+0.38	9.213	10.82
4	.004418	23172	102.37	+2.01	9.584	10.89
5	.003918	23144	90.68	−3.14	8.319	10.52
	
Mean						10.76
Standard deviation of the mean						±0.089

## Footnotes

2 Figures in brackets indicate the literature references at the end of this paper.
